# Comment to: Focal segmental glomerulosclerosis associated with mitochondrial disease by Lim et al. in Clin Nephrol Case Stud. 2017; 5: 20-25. 

**DOI:** 10.5414/CNCS109366L

**Published:** 2018-01-16

**Authors:** Josef Finsterer, Sinda Zarrouk-Mahjoub

**Affiliations:** 1 Krankenanstalt Rudolfstiftung, Vienna, Austria, and; 2 University of Tunis El Manar and Genomics Platform, Pasteur Institute of Tunis, Tunisia

**Keywords:** focal segmental glomerulosclerosis, mitochondrial disease

## Abstract

Not available.

## Letter to the Editor 

We read with interest the article by Lim et al. [1] about a 34-year-old male carrying the variant m.5538 G>A, which manifested as multisystem disease, including renal failure. We have the following comments and concerns. 

We do not agree that the variant m.5538 G>A was truly pathogenic. Heteroplasmy rate in lymphocytes was only 30%, the mutation did not segregate with the phenotype, and no biochemical investigations of muscle or fibroblasts, no single-fiber studies, and no cybrid studies had been conducted. Although MITOMAP classifies the variant as a likely pathogenic, there is only one publication cited [2]. 

Renal involvement has been repeatedly reported and includes not only focal segmental glomerulosclerosis (FSGS) and renal insufficiency, but also nephrolithiasis, nephrotic syndrome, cysts, renal tubular acidosis, Bartter-like syndrome, Fanconi syndrome, tubulointerstitial nephritis, nephrocalcinosis, and benign or malign neoplasms [3]. Did the index case present with any of these additional manifestations? Nephrolithiasis, renal failure, and renal cysts are the most frequent renal manifestations in mitochondrial disorders (MIDs) [3]. 

Obviously, the patient experienced an embolic stroke [1]. Which risk factors in addition to hypertension and diabetes were present? Was there atrial fibrillation, hyperlipidemia, heart failure, noncompaction, or smoking? Was the family history positive for cardiovascular events? 

We should be informed if heteroplasmy rate was determined also in the explanted kidney or other tissues. Did the patient undergo lactate stress testing [4]? Since immunosuppression is mitochondrion-toxic, long-term follow-up should be presented. 

Overall, this interesting report could be supplemented by proof of the pathogenicity of the accused variant, prospective investigations of organs other than the kidneys, and by more widespread family investigations. 

## Conflict of interest 

There are no conflicts of interest. 

## Funding 

No funding was received. 
